# Effect of soil contamination with fluorine on the yield and content of nitrogen forms in the biomass of crops

**DOI:** 10.1007/s11356-017-8523-6

**Published:** 2017-02-13

**Authors:** Radosław Szostek, Zdzisław Ciećko

**Affiliations:** 1 Department of Environmental Chemistry, University of Warmia and Mazury in Olsztyn, Plac Łódzki 4, 10-727 Olsztyn, Poland; 2 Faculty of Ecology, University of Ecology and Management in Warsaw, ul. Olszewska 12, 00-792 Warszawa, Poland

**Keywords:** Fluorine, Crops, Yielding, Neutralizing substances, Soil pollution

## Abstract

The research was based on a pot experiment, in which the response of eight species of crops to soil contamination with fluorine was investigated. In parallel, some inactivating substances were tested in terms of their potential use for the neutralization of the harmful influence of fluorine on plants. The response of crops to soil contamination with fluorine was assessed according to the volume of biomass produced by aerial organs and roots as well as their content of N-total, N-protein, and N-NO_3_^−^. The following crops were tested: maize, yellow lupine, winter oilseed rape, spring triticale, narrow-leaf lupine, black radish, phacelia, and lucerne. In most cases, soil pollution with fluorine stimulated the volume of biomass produced by the plants. The exceptions included grain and straw of spring triticale, maize roots, and aerial parts of lucerne, where the volume of harvested biomass was smaller in treatments with fluorine-polluted soil. Among the eight plant species, lucerne was most sensitive to the pollution despite smaller doses of fluorine in treatments with this plant. The other species were more tolerant to elevated concentrations of fluorine in soil. In most of the tested plants, the analyzed organs contained more total nitrogen, especially aerial organs and roots of black radish, grain and straw of spring triticale, and aerial biomass of lucerne. A decrease in the total nitrogen content due to soil contamination with fluorine was detected only in the aerial mass of yellow lupine. With respect to protein nitrogen, its increase in response to fluorine as a soil pollutant was found in grain of spring triticale and roots of black radish, whereas the aerial biomass of winter oilseed rape contained less of this nutrient. Among the analyzed neutralizing substances, lime most effectively alleviated the negative effect of soil pollution with fluorine. The second most effective substance was loam, while charcoal was the least effective in this respect. Our results showed the effect of soil contamination with fluorine on the yield and chemical composition of fluorine depended on the species and organ of a tested plant, on the rate of the xenobotic element and on the substance added to soil in order to neutralize fluorine.

## Introduction

Fluorine is one of the most widespread elements in the natural environment. In fact, it is the 13th most abundant element in the Earth’s crust (Ochoa-Herrera et al. 2009). The uptake of fluorine by plants from the substrate is typically low because soil-borne fluorine most often occurs in a form unavailable to plants, hence plants will absorb amounts of this element under natural conditions. However, in soils polluted with fluorine plants may take up its excessive quantities (Smolik et al. 2011).

Fluorine is a common phytotoxic air and soil pollutant (Zhang et al. 2013). Soils exposed to large emission of fluorine tend to accumulate it, which eventually has an adverse impact on agricultural production (Nowak et al. 2000). The negative effect of fluorine on plants is manifested, for example, by chlorosis (yellowing) and necrosis of leaves as well as a decreasing content of chlorophyll in leaves, the consequence of which is the inhibited growth of a plant and less biomass produced (Gupta et al. 2009). It is worth mentioning that different degrees of tolerance to fluorine compounds can be found within different plant species (Rey-Asensio and Carballeia, 2007). Moreover, hydrogen fluoride released to the environment is threefold more toxic than O_3_, SO_2,_ or NO_2_ (Weinstein and Davison 2003; Jha et al. 2008).

The influence of fluorine on plants ought to be viewed from two angles. On the one hand, the effect of this element contained in industrial gases and dusts is discussed; on the other hand, the effect of fluorine absorbed by the root system of a plant is considered (Telesiński and Śnioszek 2009). Of the two, the question of the influence of fluorine taken up by a plant through the root system is far from being thoroughly scrutinized.

In view of the above, our objective has been to determine the effect of soil contamination with fluorine on the yielding of eight crop species and on their biomass content of some nitrogen forms. The effect of soil pollution with fluorine on the analyzed characteristics of crops was examined in treatments including the application of various neutralizing substances.

## Materials and methods

### Field experiment

The study was carried out based on the eight pot experiments conducted in the years 2009–2011 at the vegetation hall of the University of Warmia and Mazury in Olsztyn. The experiments used a topsoil layer of brown soil with a granulometric composition of loamy sand. The pH of the used soil in H_2_O was 5.89; in 1 mol KCl dm^−3^, it was 4.43, and the hydrolytic acidity was 30.7 mmol(+) kg^−1^. The content of assimilable ingredients in the used soil material was as follows: for phosphorus, 43.2 mg P; for potassium, 124.5 mg K; and for magnesium, 30.0 mg Mg kg^−1^of dry mass of soil. In this soil, the following contents were also determined: organic carbon—6.0 g kg^−1^ of dry mass; total nitrogen—0.62 g kg^−1^ of dry mass; and total fluorine—125 mg kg^−1^ of dry mass.

For the inactivation of fluorine in the soil, lime, charcoal, and loam were used.

The characteristics of the basic chemical properties of the neutralizing substances used in the experiments are presented in Table 1.

**Table 1:** Chemical composition of the substances used for the inactivation of fluorine

Neutralizing substance	Element (g kg^−1^ of dry mass)					
	F _(total)_	P	K	Mg	Ca	Na
Lime (CaO)	0.50	0.12	0.75	2.55	339.21	0.09
Charcoal	2.00	0.71	9.30	2.62	7.33	0.79
Loam	0.088	0.40	21.0	17.7	23.92	7.99

### Plant material

The test plants included maize (*Zea mays* L.), yellow lupin (*Lupinus luteus* L.*)*, winter rape (*Brassica napus* L.), spring triticale (*Triticosecale Wittm.*), narrow-leafed lupin (*Lupinus angustifolius* L.), black radish (*Raphanus sativus*), phacelia (*Phacelia Juss.)*, and lucerne (*Medicago sativa* L.).

For seven species, the amount of the biomass of the above-ground parts and roots was determined, while for lucerne, only the amount of the biomass of the above-ground parts following the first mowing.

Plant growth conditions and experimental design.

In the experiments, two factors were considered. The factor of the first order included the increasing contamination of soil with fluorine in a form of potassium fluoride (the commercial form), which was used by means of simulation to contaminate the soil, while the second factor included the comparison of three substances neutralizing the soil contamination with fluorine.

Depending on the sensitivity of the tested plants, the values for the contamination of soil with fluorine amounted to the following:

- for sensitive plants, i.e., narrow-leafed lupin: 0, 20, 40, and 60 mg F kg^−1^of soil,
- for medium-sensitive plants, i.e., lucerne: 0, 50, 100, and 150 mg F kg^−1^of soil,
- for low-sensitive plants, i.e., maize, winter rape, spring triticale, black radish, and phacelia: 0, 100, 200, and 300 mg F kg^−1^of soil.

The selection of doses was guided by the average content of total fluorine in the soils of Poland according to Kabata-Pendias and Pendias (1999).

For two experiments, i.e., those involving narrow-leaved lupin and lucerne, a lower level of soil contamination with fluorine was applied as compared with the other experiments due to the fact that the leguminosae plants are more sensitive to the presence of various xenobiotics in the soil.

As regards the group of plants being sensitive to soil contamination with fluorine, yellow lupin which had been sown as an after crop following the harvest of maize was also tested. For this plant, the follow-up effect of the soil contamination with fluorine as adopted under the maize was assessed. The sensitivity of the plants to the contamination with fluorine was estimated based on the preliminary studies carried out prior to the establishment of proper pot experiments.

The following were used in the experiments as the substances neutralizing the soil contamination with fluorine: lime (at a dose equivalent to 1Hh of the soil), charcoal and loam at an amount of 3% in relation to the weight of the soil in a pot.

### Soil and nutrient treatments

In addition to the neutralizing substances, in order to satisfy the nutritional requirements of the plants, supplementary mineral fertilization with NPK was applied at the same level in all experiments. Nitrogen was applied in a form of urea at an amount of 111 mg N, phosphorus in a form of triple superphosphate 46% at an amount of 48 mg P, and potassium in a form of a 57% potassium salt at an amount equal to 111 mg K kg^−1^ of soil.

In total, each experiment included 16 objects, with three repetitions for each. The soil at an amount of 9.0 kg was thoroughly mixed with mineral fertilizers and, in the appropriate objects, with fluorine and the neutralizing substances, and then transferred to appropriately marked pots. Immediately after filling the pots with the soil with the particular components, the tested plants were sown, with 13 plants being ultimately left in a pot in all experiments, with an exception of black radish, for which 8 plants were left in a pot in each experiment.

### Harvest and measurements

During the plant vegetation, the moisture content of the soil in the pots was maintained at a level of 60% of the capillary water capacity. The plants were harvested at the stage of technological maturity; at this time, samples of plant material, divided into the above-ground parts and roots, were also taken for laboratory analyses. The biomass obtained from the pots was combined into pooled samples corresponding to the particular combinations. The samples were broken up and dried at a temperature of 60 °C.

Total nitrogen (N-tot) was determined by the Kjeldahl’s distillation method using a Speeddigester K-439 oven for mineralization and a Büchi Distillation Unit K-355 for distillation; nitrate (V) nitrogen (N-NO_3_^−^) was determined by potentiometry, using 2% acetic acid as an extraction solution (Ostrowska et al. 1991), and protein nitrogen was assayed with the Kjeldahl’s distillation method, having precipitated the proteins with trichloroacetic acid.

### Statistical analysis

The obtained study results were statistically processed using the Statistica 10.0 program and by the two-way analysis of variance (ANOVA), while the least significant differences (NIR) were determined at a level of significance *α* = 0.05 using the Duncan test (StatSoft Inc. 2010). The relationships between the fluorine contamination and the contents of calcium and magnesium in the plants were determined using the polynomial regression equations and Pearson’s simple correlation.

## Results

### Weight of aerial parts and roots of crops

The influence of increasing levels of soil contamination with fluorine on the yield and content of the analyzed nitrogen forms in crops depended on the degree of soil contamination with fluorine, the applied neutralizing substance as well as the species and organ of the test plant (Tables 2, 3, 4, and 5).

**Table 2:** Yield of aerial part and weight of roots of the analyzed plants depending on soil contamination with fluorine and neutralizing substance applied, in grams FM per pot

Soil pollution with fluorine in mg kg^−1^ of soil	Type of neutralizing substance												Mean		
	Without neutralizing substance		Lime according to 1Hh			Charcoal, 3% of soil mass			Loam, 3% of soil mass						
	Aerial mass	Roots	Aerial mass		Roots	Aerial mass		Roots	Aerial mass		Roots		Aerial mass	Roots	
Maize															
0	570.7	150.4	556.1		153.8	454.0		108.2	533.1		151.0		528.5	140.9	
100	594.3	131.1	603.9		133.8	522.6		116.2	551.7		125.6		568.1	126.7	
200	585.8	115.3	592.4		125.7	522.7		106.6	636.3		142.6		584.3	122.6	
300	594.4	135.4	614.5		144.5	502.9		119.3	588.1		137.3		575.0	134.1	
Mean	586.3	133.1	591.7		139.5	500.6		112.6	577.3		139.1		–	–	
*r*	0.66*	−0.43	0.63*		−0.22	0.38		0.28					–	–	
LSD_0.05_ for:		Aerial mass			a—22.69^; b—22.69^; a∙b—n.s.										
		Roots			a—10.92^; b—10.92^; a∙b—n.s.										
Yellow lupine															
0	28.54	4.02	30.33		4.81	30.78		4.95	31.00		4.43		30.16	4.55	
100	32.90	4.11	36.53		4.52	36.60		4.48	36.08		4.46		35.53	4.39	
200	32.69	4.74	33.98		4.86	32.24		4.79	36.09		4.61		33.75	4.75	
300	32.24	4.22	33.20		4.97	32.34		4.93	35.71		5.61		33.37	4.93	
Mean	31.59	4.27	33.51		4.79	32.99		4.79	34.70		4.78		–	–	
*r*	0.45	0.24	0.22		0.16	0.01		0.06	0.40		0.60*		–	–	
LSD_0.05_ for:		Aerial mass			a—2.72^; b—n.s.; a∙b—n.s.										
		Roots			a—n.s.; b—n.s.; a∙b—n.s.										
Winter oilseed rape															
0	44.76	3.27	61.32		4.46	63.25		5.02	59.28		4.28		57.15	4.27	
100	60.44	4.09	86.23		4.94	71.88		5.98	68.42		4.83		71.74	4.96	
200	73.36	4.79	87.19		4.92	83.20		5.96	84.22		5.88		81.99	5.39	
300	72.53	5.16	75.89		5.56	77.78		6.21	72.39		6.03		74.65	5.74	
Mean	62.77	4.33	77.66		4.97	74.03		5.79	71.08		5.25		–	–	
*r*	0.84**	0.83**	0.43		0.79**	0.73**		0.69*	0.57*		0.84**		–	–	
LSD_0.05_ for:		Aerial mass			a—5.30^; b—5.30^; a∙b—n.s.										
		Roots			a—0.38^; b—0.38^; a∙b—n.s.										
Narrow-leaf lupine															
0	48.94	12.40	24.45		6.37	81.21		19.85	84.97		19.91		59.89	14.63	
20	78.34	17.12	49.06		12.74	78.45		18.33	75.49		16.84		70.33	16.26	
40	66.55	16.48	55.68		15.28	71.26		20.98	75.06		16.50		67.14	17.31	
60	69.54	20.06	51.18		13.99	68.93		18.79	78.68		18.95		67.08	17.95	
Mean	65.84	16.51	45.09		12.09	74.96		19.49	78.55		18.05		–	–	
*r*	0.42	0.81**	0.73**		0.78**	−0.52		−0.02	−0.30		−0.13		–	–	
LSD_0.05_ for:		Aerial mass			a—7.06^; b—7.06^; a∙b—14.12^										
		Roots			a—2.23^; b—2.23^; a∙b—4.46^										
Black radish															
0	189.3	83.2		192.7	83.0		193.6	82.0		195.4	93.2		192.7	85.3	
100	193.5	83.8		207.4	100.4		197.0	97.8		202.5	102.6		200.1	96.1	
200	192.2	86.8		217.3	102.6		208.2	100.5		213.0	109.8		207.7	99.9	
300	204.7	87.8		221.9	101.5		210.4	101.9		218.6	108.5		213.9	99.9	
Mean	194.9	85.4		209.8	96.9		202.3	95.5		207.4	103.5		–	–	
*r*	0.67*	0.37		0.73**	0.70**		0.81**	0.77**		0.76**	0.73**		–	–	
LSD_0.05_ for:		Aerial mass			a—7.12^; b—7.12^; a∙b—n.s.										
		Roots			a—4.66^; b—4.66^; a∙b—n.s.										
Phacelia															
0	207.72	6.65		224.30	6.60		196.45	5.42		213.63	4.99		210.52	5.91	
100	209.50	7.60		228.39	7.86		217.15	6.71		238.63	7.62		223.42	7.45	
200	207.80	7.63		220.85	8.19		213.26	6.81		224.37	6.09		216.57	7.18	
300	206.95	8.31		217.35	7.07		207.61	8.38		222.77	6.36		213.67	7.53	
Mean	207.99	7.55		222.72	7.43		208.62	6.83		224.85	6.26		**–**	**–**	
*r*	−0.10	0.54		−0.29	0.22		0.32	0.80**		0.12	0.28		**–**	**–**	
LSD_0.05_ for:		Aerial mass			a—7.72^; b—7.72^; a∙b—n.s.										
		Roots			a—0.69^; b—0.69^; a∙b—n.s.										
Spring triticale															
–	Seed	Straw	Roots	Seed	Straw	Roots	Seed	Straw	Roots	Seed	Straw	Roots	Seed	Straw	Roots
0	40.54	44.96	21.63	32.88	43.07	15.20	27.63	39.52	12.17	35.90	39.31	14.20	34.24	41.72	15.80
100	35.82	43.36	19.57	32.82	42.36	15.37	29.05	38.60	13.33	30.87	38.91	14.83	32.14	40.81	15.78
200	30.30	42.51	19.03	30.82	41.88	15.43	28.36	37.69	14.00	28.60	38.35	18.70	29.52	40.11	16.79
300	26.61	41.49	18.63	29.95	41.15	15.43	26.78	36.95	14.33	28.11	38.12	22.47	27.86	39.43	17.72
Mean	33.32	43.08	19.72	31.62	42.12	15.36	27.96	38.19	13.46	30.87	38.67	17.55	**–**	**–**	**–**
*r*	−0.94**	−0.67*	−0.64*	−0.43	−0.34	0.07	−0.15	−0.47	0.67*	−0.70**	−0.20	0.96**	**–**	**–**	**–**
LSD_0.05_ for:		Seed			a—2.33^; b—2.33^; a∙b—4.66^										
		Straw			a—n.s.; b—1.88^; a∙b—n.s.										
		Roots			a—1.01^; b—1.01^; a∙b—2.03^										
Alfalfa															
0	61.07	–		68.56	–		61.80	–		59.53	–		62.74	–	
50	53.65	–		59.93	–		57.45	–		56.87	–		56.98	–	
100	46.31	–		53.42	–		49.50	–		50.42	–		49.91	–	
150	37.91	–		42.82	–		40.94	–		39.37	–		40.26	–	
Mean	49.74	–		56.18	–		52.42	–		51.55	–		–	–	
*r*	−0.89**	–		−0.91**	–		−0.89**	–		−0.89**	–		–	–	
LSD_0.05_ for:		Aerial mass			a—3.89^; b—3.89^; a∙b—n.s.										

LSD (least significant difference) for: a—F soil contamination; b—neutralizing substance; a∙b—interaction

*Correlation coefficient (*r*) significant for *α* = 0.05; **correlation coefficient (*r*) significant for *α* = 0.01; ^significant for 0.05

**Table 3:** Concentration of total nitrogen in analyzed plants depending on soil contamination with fluorine and neutralizing substance applied, in grams N per kilogram DM

Soil pollution with fluorine in mg kg^−1^ of soil	Type of neutralizing substance												Mean		
	Without neutralizing substance			Lime according to 1Hh			Charcoal, 3% of soil mass			Loam, 3% of soil mass					
	Aerial mass	Roots		Aerial mass	Roots		Aerial mass		Roots	Aerial mass		Roots	Aerial mass		Roots
Maize															
0	6.7	6.3		7.4	5.7		8.2		6.3	7.4		6.8	7.4		6.3
100	7.4	6.3		7.8	6.8		8.5		7.6	7.4		6.8	7.8		6.9
200	7.4	6.8		7.9	6.8		8.6		7.9	7.7		6.7	7.9		7.0
300	7.3	6.2		7.9	6.3		8.5		7.4	7.7		6.3	7.8		6.5
Mean	7.2	6.4		7.7	6.4		8.4		7.3	7.5		6.6	**–**		**–**
*r*	0.63*	0.09		0.77**	0.44		0.31		0.60*	0.66*		−0.74**	**–**		**–**
LSD_0.05_ for:		Aerial mass			a—0.20^; b—0.20^; a∙b—n.s.										
		Roots			a—0.18^; b—0.18^; a∙b—0.36^										
Yellow lupine															
0	38.7	15.1		36.5	17.8		36.8		16.6	37.6		17.0	37.4		16.6
100	40.4	16.3		35.9	16.9		38.4		16.4	37.6		15.7	38.1		16.3
200	35.5	18.1		35.4	16.4		36.3		15.7	36.7		15.0	36.0		16.3
300	35.4	16.9		35.4	16.5		34.3		14.6	36.3		14.8	35.3		15.7
Mean	37.5	16.6		35.8	16.9		36.5		15.8	37.0		15.6	**–**		**–**
*r*	−074**	0.63*		−0.62*	−0.56		−0.53		–0.74**	−0.60*		−0.76	**–**		**–**
LSD_0.05_ for:		Aerial mass			a—0.89^; b—0.89^; a∙b—1.77^										
		Roots			a—0.39^; b—0.39^; a∙b—0.78^										
Winter oilseed rape															
0	45.3	28.8		48.0	30.2		43.1		33.0	44.2		28.8	45.1		30.2
100	45.7	37.6		47.7	36.2		43.5		39.1	42.0		36.2	44.7		37.3
200	45.3	30.6		46.9	32.3		43.6		34.2	42.0		32 .8	44.5		32.5
300	45.0	30.2		45.8	31.7		43.2		34.2	41.6		31.7	43.9		32.0
Mean	45.3	31.8		47.1	32.6		43.3		35.1	42.4		32.4	**–**		**–**
*r*	−0.17	−0.09		−0.76**	0.03		0.03		−0.05	−0.68*		0.22	**–**		**–**
LSD_0.05_ for:		Aerial mass			a—n.s.; b—1.08^; a∙b—n.s.										
		Roots			a—0.83^; b—0.83^; a∙b—n.s.										
Narrow-leaf lupine															
0	47.1	23.0		42.5	23.0		34.4		24.0	40.4		21.4	41.4		22.8
20	47.2	24.0		42.4	23.7		35.4		23.1	41.7		22.6	41.7		23.3
40	47.7	24.3		42.8	23.8		35.1		23.2	41.4		23.1	41.7		23.6
60	47.1	24.3		42.7	23.5		34.0		23.1	41.1		22.5	41.2		23.3
Mean	47.2	23.9		42.6	23.5		34.7		23.3	41.1		22.4	**–**		**–**
*r*	0.06	0.68*		0.18	0.39		−0.12		−0.30	0.22		0.57*	**–-**		**–**
LSD_0.05_ for:		Aerial mass			a—n.s.; b—0.94^; a∙b—n.s.										
		Roots			a—n.s.; b—0.57^; a∙b—n.s.										
Black radish															
0	14.4	13.8		13.7	13.9		14.4	13.7		14.4	13.7		14.2	13.8	
100	17.1	14.4		14.1	14.0		14.7	15.3		18.4	15.1		16.1	14.7	
200	20.4	15.8		17.4	15.1		21.4	16.4		19.1	14.9		19.6	15.5	
300	20.4	15.7		20.7	16.1		21.1	16.4		18.4	13.9		20.1	15.5	
Mean	18.1	14.9		16.5	14.8		17.9	15.4		17.6	14.4		**–**	**–**	
*r*	0.94**	0.88**		0.96**	0.93**		0.87**	0.81**		0.75**	0.07		**–**	**–**	
LSD_0.05_ for:		Aerial mass			a—0.49^; b—0.49^; a∙b—0.99^										
		Roots			a—0.39^; b—0.39^; a∙b—0.78^										
Phacelia															
0	31.1	20.4		31.1	21.7		31.7	20.4		34.0	18.4		32.0	20.2	
100	33.1	20.6		31.1	21.7		30.2	20.4		33.7	18.4		32.0	20.3	
200	33.4	21.3		31.7	20.4		28.8	21.1		33.1	20.1		31.8	20.7	
300	33.7	21.1		32.0	19.7		26.4	20.4		32.4	19.7		31.1	20.2	
Mean	32.8	20.8		31.5	20.9		29.3	20.6		33.3	19.1		**–**	**–**	
*r*	0.76**	0.59*		0.63*	−0.88**		−0.87**	0.43		−0.69*	0.57*		**–**	**–**	
LSD_0.05_ for:		Aerial mass			a—n.s.; b—0.74^; a∙b—1.48^										
		Roots			a—n.s.; b—0.51^; a∙b—1.02^										
Spring triticale															
–	Seed	Straw	Roots	Seed	Straw	Roots	Seed	Straw	Roots	Seed	Straw	Roots	Seed	Straw	Roots
0	19.1	5.3	4.0	22.4	6.0	6.0	21.4	6.0	5.3	20.2	6.7	3.7	20.8	6.0	4.8
100	21.7	7.3	4.4	24.5	6.7	4.4	21.7	8.7	4.4	22.9	8.7	5.7	22.7	7.9	4.7
200	22.4	7.3	7.1	25.4	8.0	5.7	23.1	7.3	4.8	23.0	7.3	4.4	23.5	7.5	5.5
300	23.1	7.3	7.1	26.4	8.0	7.7	23.5	6.7	6.0	22.8	6.0	7.7	23.9	7.0	7.1
Mean	21.6	6.8	5.7	24.7	7.2	6.0	22.4	7.2	5.1	22.2	7.2	5.4	**–**	**–**	**–**
*r*	0.91**	0.77**	0.86**	0.95**	0.94**	0.61*	0.68*	0.08	0.44	0.71**	−0.39	0.78**	**–**	**–**	**–**
LSD_0.05_ for:		Seed			a—0.57^; b—0.57^; a∙b—n.s.										
		Straw			a—0.18^; b—0.18^; a∙b—0.36^										
		Roots			a—0.13^; b—0.13^; a∙b—0.27^										
Alfalfa															
0	25.2	–		24.1	–		25.6	–		26.5	–		25.3	–	
50	27.4	–		24.9	–		26.2	–		26.6	–		26.3	–	
100	27.8	–		25.5	–		26.9	–		26.6	–		26.7	–	
150	28.3	–		26.9	–		26.2	–		26.9	–		27.1	–	
Mean	27.1	–		25.3	–		26.2	–		26.6	–		–	–	
*r*	0.84**	–		0.93**	–		0.25	–		0.26	–		–	–	
LSD_0.05_ for:		Aerial mass			a—0.66^; b—0.66^; a∙b—1.31^										

LSD (least significant difference) for: a—F soil contamination; b—neutralizing substance; a∙b—interaction

*Correlation coefficient (*r*) significant for *α* = 0.05; **correlation coefficient (*r*) significant for *α* = 0.01; ^significant for 0.05

**Table 4:** Concentration of protein nitrogen in analyzed plants depending on soil contamination with fluorine and neutralizing substance applied, in grams N kilogram DM

Soil pollution with fluorine in mg kg^−1^ of soil	Type of neutralizing substance				Mean
	Without neutralizing substance	Lime according to 1 Hh	Charcoal, 3% of soil mass	Loam, 3% of soil mass	
Maize aerial mass					
0	4.9	5.1	6.1	5.2	5.3
100	5.5	5.3	6.3	5.2	5.6
200	5.5	5.4	6.4	5.3	5.7
300	5.4	5.4	6.3	5.4	5.6
Mean	5.3	5.4	6.3	5.3	–
*r*	0.55	0.62*	0.47	0.69*	–
LSD_0.05_ for: a—0.15^; b—0.15^; a∙b—n.s.					
Yellow lupine aerial mass					
0	25.7	27.1	28.0	25.3	26.5
100	26.2	26.8	28.7	26.8	27.1
200	25.9	26.7	27.0	26.7	26.6
300	25.1	26.6	25.8	26.7	26.1
Mean	25.7	26.8	27.4	26.4	–
*r*	−0.08	−0.68*	−0.85**	0.72**	–
LSD_0.05_ for: a—0.24^; b—0.24; a∙b—0.48					
Winter oilseed rape aerial mass					
0	34.0	30.7	30.3	32.1	31.8
100	32.4	30.7	30.8	29.4	30.8
200	31.7	29.5	30.8	28.1	30.0
300	31.5	28.8	29.1	27.9	29.3
Mean	32.4	29.9	30.3	29.4	–
*r*	−0.94**	−0.94**	−0.55	−0.93**	–
LSD_0.05_ for: a—0.14^; b—0.14^; a∙b—0.29					
Narrow-leaf lupine aerial mass					
0	31.9	28.8	23.7	24.2	27.2
20	31.6	28.6	24.0	25.4	27.4
40	31.7	27.8	23.9	26.2	27.4
60	31.7	27.0	23.6	25.6	27.0
Mean	31.7	28.1	23.8	25.4	–
*r*	−0.35	−0.97**	−0.10	0.77**	–
LSD_0.05_ for: a—0.14^; b—0.14^; a∙b—0.28^					
Black radish roots					
0	11.3	11.2	11.1	11.3	11.2
100	11.7	11.5	11.9	12.4	11.9
200	13.2	12.1	13.3	12.2	12.7
300	12.0	12.8	13.2	11.4	12.4
Mean	12.1	11.9	12.4	11.8	–
*r*	0.57*	0.97**	0.93**	0.00	–
LSD_0.05_ for: a—0.12^; b—0.12^; a∙b—0.24					
Phacelia aerial mass					
0	25.1	24.6	24.4	24.5	24.7
100	25.8	24.7	23.8	24.3	24.7
200	26.0	25.0	22.8	23.7	24.4
300	26.1	25.2	20.9	23.3	23.9
Mean	25.8	24.9	23.0	24.0	–
*r*	0.88**	0.93**	−0.97**	−0.97**	–
LSD_0.05_ for: a—0.12^; b—0.12; a∙b—0.24					
Spring triticale seed					
0	17.2	19.2	18.9	17.8	18.3
100	19.5	19.4	19.3	20.1	19.6
200	20.2	21.6	20.9	20.3	20.8
300	20.8	21.9	21.3	19.7	20.9
Mean	19.4	20.5	20.1	19.5	–
*r*	0.94**	0.94**	0.96**	0.65*	–
LSD_0.05_ for: a—0.13^; b—0.13^; a∙b—0.26					
Alfalfa aerial mass					
0	20.6	19.8	19.9	20.9	20.3
50	21.7	20.5	20.1	21.1	20.9
100	22.0	21.0	20.5	20.8	21.1
150	22.3	22.0	20.1	20.7	21.3
Mean	21.7	20.8	20.2	20.9	–
*r*	0.93**	0.99**	0.40	−0.82**	–
LSD_0.05_ for: a—0.12^; b—0.12^; a∙b—0.25^					

SD (least significant difference) for: a—F soil contamination; b—neutralizing substance; a∙b—interaction

*Correlation coefficient (*r*) significant for *α* = 0.05; **correlation coefficient (*r*) significant for *α* = 0.01; ^significant for 0.05

**Table 5:** Concentration of nitrate (V) nitrogen (NO_3_^−^) in analyzed plants depending on soil contamination with fluorine and neutralizing substance applied, in milligram NO_3_^−^ per kilogram^−1^ DM

Soil pollution with fluorine in mg kg^−1^ of soil	Type of neutralizing substance				Mean
	Without neutralizing substance	Lime according to 1Hh	Charcoal, 3% of soil mass	Loam, 3% of soil mass	
Maize aerial mass					
0	205	219	377	301	276
100	233	267	370	315	296
200	329	308	353	342	333
300	267	353	335	387	335
Mean	258	287	359	336	–
*r*	0.68*	0.91**	−0.94**	0.92**	–
LSD_0.05_ for: a—19.29^; b—19.29^; a∙b—38.59					
Yellow lupine aerial mass					
0	2497	2407	2461	2265	2407
100	2488	2390	2490	2247	2403
200	2479	2372	2484	2211	2386
300	2443	2318	2479	2207	2362
Mean	2477	2372	2478	2233	–
*r*	−0.79**	−0.63*	0.24	−0.53	–
LSD_0.05_ for: a—n.s.^; b—43.35; a∙b—n.s.					
Winter oilseed rape aerial mass					
0	2472	3131	1957	1814	2343
100	2615	2882	2046	1797	2335
200	2401	2579	2099	1779	2214
300	2295	2526	2134	2063	2255
Mean	2446	2779	2059	1863	–
*r*	−0.68*	−0.96**	0.88**	0.69*	–
LSD_0.05_ for: a—50.80^; b—50.80^; a∙b—101.61					
Narrow-leaf lupine aerial mass					
0	1923	1868	1571	1819	1795
20	1985	1906	1861	1861	1903
40	2357	1989	2378	2212	2234
60	1737	1836	1571	1737	1720
Mean	2000	1900	1845	1907	–
*r*	−0.07	−0.02	0.15	0.35	–
LSD_0.05_ for: a—197.18^; b—n.s.^; a∙b—n.s.					
Black radish roots					
0	482	482	489	517	492
100	506	489	517	535	512
200	514	492	535	553	523
300	446	499	535	482	490
Mean	487	490	519	522	–
*r*	−0.38	0.52	0.58*	−0.33	–
LSD_0.05_ for: a—24.55^; b—24.55^; a∙b—n.s.					
Phacelia aerial mass					
0	1761	1797	2046	2063	1917
100	1783	1850	1885	1636	1789
200	1814	2099	1814	1121	1712
300	1957	2001	1792	1103	1713
Mean	1829	1937	1884	1481	–
*r*	0.82**	0.78**	−0.93**	−0.95**	–
LSD_0.05_ for: a—40.01^; b—40.01; a∙b—80.02					
Spring triticale seed					
0	736	667	736	736	719
100	787	669	753	719	732
200	821	671	821	650	741
300	753	656	616	621	662
Mean	774	666	732	682	–
*r*	0.23	−0.33	−0.41	−0.92**	–
LSD_0.05_ for: a—33.47^; b—33.47^; a∙b—66.95					
Alfalfa aerial mass					
0	418	472	565	736	548
50	445	500	572	744	565
100	479	685	702	753	655
150	582	736	616	787	680
Mean	481	598	614	755	–
*r*	0.91**	0.92**	0.54	0.85**	–
LSD_0.05_ for: a—31.99^; b—31.99^; a∙b—63.98^					

LSD (least significant difference) for: a—F soil contamination; b—neutralizing substance; a∙b—interaction

*Correlation coefficient (*r*) significant for *α* = 0.05; **correlation coefficient (*r*) significant for *α* = 0.01; ^significant for 0.05

In general, the soil contamination with fluorine stimulated the amount of biomass harvested from the test crops.

The current study suggests that maize should be classified as a plant particularly resistant to soil contamination with fluoride. The aerial biomass of this crop gradually increased in volume as the soil contamination with fluoride was intensified. In the series without any neutralizing substance, the amount of aerial biomass of maize in the control treatment was 570.7 g pot^−1^ FM, being the smallest of all the control treatments. Under the influence of the lowest dose of fluorine, a 4% increase in the amount of biomass compared to its quantity in the control was observed. As for the roots, a reverse relationship occurred, that is the mass of roots decreased in response to the soil pollution with fluorine, so that in the treatment with the medium dose of this xenobiotic the root biomass corresponded to 23% of the root biomass from the control. The substances added to soil for fluorine inactivation had various effects on the amount of harvested biomass. For example, charcoal produced an unquestionably negative effect on the amount of maize biomass. After it had been introduced to soil, the mass of aerial maize organs decreased by an average of 15% and the root biomass was 16% lower than in the control. Of the three neutralizing substances, lime had the most positive effect on the amount of maize biomass. The reason was most probably the higher soil reaction, which resulted in a more limited availability of fluorine to plants. With respect to roots, when all the three contamination alleviating substances were compared, charcoal proved to be the least effective. Although the application of this substance contributed to a slight increase of the maize root biomass, such as by 7 and 15% in the pots polluted with 100 and 300 mg F kg^−1^ of soil, respectively, the biomass harvested from these treatments continued to be lower than in the parallel treatments without a neutralizing substance. The mass of maize roots in the control treatment from that series was the smallest of all the control variants of the experiment and equalled 108.2 g pot^−1^ FM.

Our research showed a positive effect of soil contamination with fluorine on the quantity of biomass obtained from yellow lupine. In the series without any neutralizing substance, the yield of both green biomass and roots of yellow lupine was higher in response to the growing doses of fluorine. Regarding the biomass of aerial organs, its highest volume was produced by plants growing in a pot contaminated with the lowest dose of fluorine, i.e., 100 mg F kg^−1^ of soil, where it was 15% higher than the amount of biomass harvested from the control. With respect to root biomass, its highest quantity in the same series was recorded in the treatment polluted with the middle dose of fluorine, i.e., 200 mg F kg^−1^ of soil, where it was 18% higher than the mass of roots from the control variant.

In the individual series which involved the application of a neutralizing substance, the average amount of aerial and root biomass from yellow lupine was higher than the amount of biomass harvested from the series without any neutralizing substances, which might confirm the effectiveness of their application for the purpose of inactivating fluorine. In brief, the aerial biomass was most positively affected by loam, and the least modified by charcoal. The positive effect of loam manifesting by a more limited influence of fluorine on the yield of yellow lupine aerial biomass was most probably the consequence of the close affinity of fluorine to aluminum, which is easily bound by this mineral via the anion exchange path. Besides, if soil contains large quantities of calcium and magnesium, these elements can also contribute to the binding of fluorine into hardly dissolvable compounds. On the other hand, our analysis of the average mass of yellow lupine roots obtained in the series with the contribution of lime, charcoal, or loam shows similar mean values, oscillating around 4.79 g pot^−1^ FM. The mass of roots obtained in the series without any neutralizing substance was lower by an average of 11%. Thus, the results may indicate that all the applied fluorine-inactivating substances had a positive effect. In our experiment, yellow lupine was a catch crop, which may explain the higher resistance of this plant to the soil contamination with fluorine.

In our study, winter oilseed rape proved to be resistant to fluorine, as the biomass of aerial parts and roots of this plant increased significantly in all the experimental series while the degree of soil contamination with fluorine grew more severe. Thus, in the series without any neutralizing substance and in the treatment polluted with 300 mg F kg^−1^ of soil, the mass of the aerial parts and roots increased by 62 and 58%, respectively, compared to the control. In addition, a big difference was documented between the amount of the biomass of aerial parts and that of roots of winter oilseed rape. In fact, the aerial biomass amount was 14-fold larger than that of roots. Also, the current research showed that each of the tested neutralizing substances added to soil resulted in a significant increase in the amount of biomass of both aerial parts and roots. Our analysis of the average quantity of aerial biomass obtained from winter oilseed rape proved that lime had the most beneficial effect on the yield and development of this plant, while charcoal was the second most effective substance and loam was the least effective one in this regard. The average amounts of biomass obtained in the individual series of this experiment were higher than the average biomass harvested from pots without any neutralizing substance by 24, 18, and 13%. The above is supported by agricultural practice, in which liming is most often recommended to use for neutralization of polluted soils. On the other hand, the toxic effect of soil contamination with fluorine was most strongly inhibited by the soil incorporation of charcoal.

Another plant submitted to our investigation was narrow-leaf lupine, which responded to the soil pollution with fluorine by increasing its biomass. This direction of change concerned both aerial and root biomass in the series with no neutralizing substance and in the ones where liming was performed. And although in the other two series, with charcoal and with loam, a negative relationship appeared between the increasing doses of fluorine and the amount of biomass from aerial organs and roots of narrow-leaf lupine, the average amount of biomass obtained in these series was higher than the amount of biomass in the control series. In the trials with narrow-leaf lupine, the soil pollution with fluorine was fivefold lower in degree than in the trials with most of the tested plants. It is therefore plausible that the amount of harvested biomass would have been much smaller if the level of soil contamination with fluorine had corresponded to the degrees tested under the other plants.

Black radish distinguished itself among the tested plants by being most resistant to soil pollution with fluorine. As the soil pollution level with this xenobiotic increased, the biomass of black radish increased significantly in all the series of the experiment, both in terms of aerial organs and roots. In the series with no neutralizing substance, the highest dose of fluorine contributed to an 8 and 6% increase of the aerial and root biomass, respectively, versus the control. Also, a positive influence of the applied neutralizing substances was demonstrated, which was verified by the average amounts of biomass from the individual series, which were higher than the amount of biomass from the control series. Regarding the aerial biomass, lime had the best effect, while the roots of black radish were most positively affected by loam.

Another test plant was phacelia, which typically responded to the soil contamination with fluorine by increasing the amounts of biomass of both aerial organs and roots. However, the aerial biomass in the series with no neutralizing substance and in the limed series slightly decreased in response to the increasing doses of fluorine introduced to soil. All the neutralizing substances had a positive influence on the amount of aerial biomass of phacelia, with loam being most stimulating and charcoal producing the weakest effect. Concerning the roots of this plant, the increasing soil contamination with fluorine contributed to an increase in their biomass in all series of the experiment. In the pots polluted with the lowest dose of fluorine, i.e., 100 mg F kg^−1^ of soil, the highest increase in the phacelia root biomass versus the control was observed in the series with loam. When the highest fluorine dose (300 mg F kg^−1^ of soil) was applied, the highest increase in the root mass ocurred in the series with charcoal. Although the positive effect of soil contamination with fluorine was confirmed in all the series of this experiment, the applied soil additives generally had a negative influence on the amount of root biomass produced by this plant. This is indicated by the amounts of root biomass obtained in the control treatments of the individual series as well as the average amounts of root biomass from all combinations of these series. Thus, the average quantities of root biomass obtained in the experimental series were as follows: 7.55 g pot^−1^ FM in the series with no neutralizing substance, 7.43 g pot^−1^ FM in the series with lime, 6.83 g pot^−1^ FM in the series with charcoal and 6.26 g pot^−1^ FM in the series with loam. These data suggest that the negative effect of fluorine on the amount of the phacelia root biomass was most effectively buffered in the series with lime and the least effectively suppressed in the series with loam.

In our experiment, spring triticale responded to the growing soil contamination with fluorine by decreasing the biomass of the three analyzed plant parts, except roots in the series with charcoal and with loam. The organ of spring triticale which proved to be most sensitive to fluorine pollution was its grain. In the series with no neutralizing substance, the increasing soil pollution with fluorine cased a gradual decline in the gran mass, which in the treatment with the highest dose of fluorine, i.e., 300 mg F kg^−1^ of soil, was the biggest and reached 34% relative to the control in this series. In turn, the amount of biomass of straw decreased in the range of 4 to 8% compared to the control treatment in this series. Recapitulating the results pertaining to the spring tritical biomass, it should be concluded that none of the applied neutralizing substances was able to limit the negative effect of fluorine on the biomass of this crop.

Lucerne is another test plant which should be considered as a particularly sensitive one to soil pollution with fluorine. This plant showed the earliest symptoms of response to soil contamination with fluorine at the plant emergence stage. Their severe dwarfism was a characteristic sign of the negative effect of fluorine on lucerne plants. With respect to the first cut of lucerne as well as the later growth stages, distinct growth inhibition of this plant was observable. In the series with no neutralizing substance, the amount of the aerial biomass in the control was 61.07 g pot^−1^ FM. As the soil contamination with fluorine increased, the amount of the aerial biomass gradually decreased, so that in the treatment with the highest fluorine dose, i.e., 150 mg F kg^−1^ of soil, this decrease was the biggest, reaching 38% relative to the control. The application of the substances expected to neutralize fluorine did not alter the negative dependence between the harvested biomass and the degree of soil contamination with this element, even though they limited the negative effect of fluorine on the amount of the biomass of aerial organs in nearly every treatment. Our comparison of the average amounts of aerial biomass reveals that the aerial biomass of the first cut of lucerne was most positively affected by lime, followed by charcoal, while loam had the weakest influence.

It should be borne in mind that in the treatments with lucerne, the soil contamination with fluorine was twofold lower than in the case of most of the other plants, implicating that if the soil had been polluted up to the level set for most plants, the decrease in the biomass amount would have been greater.

It is worth noticing that the neutralizing substances tested in our research, added to soil in order to inactivate fluorine, generally limited the negative effect of this xenobiotic on the harvested biomass of the test crops, but their effectiveness differed from plant to plant.

### Concentration of nitrogen forms in the biomass of crops

The influence of fluorine on plants also involves some modification of their chemical composition, which may deteriorate their quality (Tables 3, 4, and 5).

The total nitrogen content in particular plant organs depended on the soil pollution with fluorine, soil enrichment with neutralizing substances and plant species. The total nitrogen content was higher in the aerial parts than in roots of all the plants. Its highest average content was determined in aerial organs of winter oilseed rape (44.6 g N kg^−1^ DM), narrow-leaf lupine (41.5 g N kg^−1^ DM), and yellow lupine (36.7 g N kg^−1^ DM), while the lowest amount of total nitrogen was detected in roots of spring triticale (5.5 g N kg^−1^ DM). Soil contamination with fluorine contributed to an increase in the N-total content in grain and roots of spring triticale, aerial mass, and roots of black radish and in aerial mass of lucerne.

Soil contamination with fluorine as well as the substances introduced to soil in order to inactivate the pollutant were found to affect the protein nitrogen content in usable plant organs. Its average share in total nitrogen was from 65.8% in narrow-leaf lupine to 87.6% in spring triticale. Its highest content was determined in aerial mass of oilseed rape, while the lowest one was detected in aerial mass of maize. The average content of protein nitrogen in these plants and organs was 30.5 and 5.6 g N kg^−1^ DM, respectively. The increasing pollution of soil with fluorine contributed to an increase in the protein nitrogen in grain of spring triticale and in roots of black radish. A reverse relationship, i.e., a decrease in the content of this nitrogen form parallel to the increasing soil contamination with fluorine was observed in aerial mass of winter oilseed rape. The substances applied to soil in order to inactivate fluorine generally decreased the content of N-protein.

## Discussion

The literature contains data indicating diverse effects of soil contamination with fluorine on the harvested biomass of crops. Our results seem to verify these findings, as some of our data coincide with the references while others are completely divergent.

In their experiment on maize, Cui et al. (2011) noted an 85% decrease of biomass versus the control in response to the highest dose of fluorine, which was 1500 mg F kg^−1^ of soil. Kumar and Singh (2015) also demonstrated a 73% decline in the amounts of roots biomass harvested from *Gossypium hirsutum* L. under the irrigation water contamination with fluorine in a dose of 1000 ppm. Telesiński et al. (2012) conducted an experiment on common wheat and white mustard, in which they demonstrated that the only dose of fluorine which stimulated significantly the growth of these plants was 1 mg F kg^−1^ of soil. Higher doses of fluorine, i.e., 10, 100, and 1000 mg F kg^−1^ of soil, had a negative effect on the volume of biomass of these plants. Similar results were reported by Saini et al. (2012), who observed a 20% decrease in the biomass of *Prosopis juliflora* growing in soil polluted with a dose of fluorine equal 100 mg F kg^−1^ of soil. Results reported by Elloumi et al. (2005) from their experiment on *Amygdalis communis* or Chakrabarti et al. (2012) who conducted trials on *Cicer arietinum*, where the highest dose of fluorine resulted in an 80% decrease in the mass of seeds from this plant. Stevens et al. (1998a, 1998b) demonstrated negative correlations between the soil contamination with fluorine and the biomass obtained from tomato and oat. Joshi and Bhardwaj (2012) showed that the lowest dose of fluorine caused a decrease in the biomass of common wheat aerial organs and roots by 11 and 16%, respectively, compared to the control. Telesiński et al. (2012) demonstrated that a dose of 441 mg F kg^−1^ of soil in a trial with white mustard as well as a dose of 503 mg F kg^−1^ of soil in a trial with common wheat contributed to a 50% decrease in the mass of roots of these plants. The negative influence of sodium fluoride on bean germination was shown by Yu (1996). Datta et al. (2012) noticed that the mass of chickpea germinated on Petri plates decreased by 75 and 32% in response to doses of fluorine of 0.1 and 4.0 mM, respectively. Gadi et al. (2012) reported that the highest dose of fluorine such as 1 mM depressed the germination of bean seeds by 18% compared to the control. Yamauchi et al. (2000) reported that doses of fluorine in the range of 5 to 30 mg F kg^−1^ of soil did not affect negatively the growth and development of seed rice. The adverse effect of this xenobiotic resulting in a decreased biomass of rice was not manifested until higher doses of fluorine, such as 50 and 100 mg F kg^−1^ of soil, were applied. Elrashidi et al. (1998) demonstrated experimentally that a dose of 100 mg F kg^−1^ of soil caused a significantly negative effect on the biomass of spring barley. The report by Pant et al. (2008) points to the toxic influence of fluorine on such plants as mustard green, chickpea, common wheat, and tomatoes. Bhargava and Bhardwaj (2010) noted that common wheat kernel germination decreased by 88% at a dose of 20 mg F dm^−3^ and the biomass of this crop was 21% lower than in the control treatment.

The reactions of plants to soil contamination with fluorine are complex and involve changes in many biochemical processes associated with content of nitrogen forms in the biomass of crops.

The content of total nitrogen reported by Wulff and Kärenlampi (1996) in the dry matter of needles of common pine (*Pinus sylvestris* L.) and spruce (*Picea abies* L.) exposed to fluorine was 24 and 8% higher, respectively, than in the control. Also, a study by Holopainen et al. (1991) on common spruce suggested a positive effect of the fluorine pollution on the total nitrogen content in needles of this tree species. The increase was 13% relative to the control, and the content of nitrogen in the analyzed objects ranged around 13.1 g N kg^−1^ DM. In our experiment, a decrease in the content of this element was determined only in the aerial mass of yellow lupine, which could be attributed to the negative effect of fluorine pollution on the protein synthesis in this plant.

Literature contains very modest information regarding the effect of fluorine on the content of individual forms of nitrogen in plants. Most researchers discuss only the total protein content in plants. At this point, the following can be cited: Asthir et al. (1999) testing chickpea (*C. arietinum* L.), or Saleem et al. (2015), experimenting with potato, who observed an increasing total protein content under the influence of soil contamination with fluorine. In their experiment on mulberry, Rao et al. (2013) detected a 59% decrease in the total protein content in leaves. Fluorine was also found to be responsible for a decrease in the total protein content in 13 plant species tested by Pal et al. (2012). Gadi et al. (2012) report a lowered total protein content in Mung bean (*Vigna radiata* L.), in which the lowest tested doses of fluorine equal 1 mM reduced the total protein content by 43%. In their experiment on water fern (*Azolla filiculoides* L.), Eyini et al. (1999) observed a 31% decrease in the total protein content at the level of fluorine pollution equal 50 ppm. Declining total protein concentrations resulting from the influence of fluorine can be explained by the depressed synthesis of this nutrient, its accelerated degradation and the use of protein for processes which are activated to obtain energy in the presence of the metabolic stress induced by fluorine.

In most cases, the substances applied in our experiment to inactivate fluorine contributed to a slight increase in the total protein content. Dey et al. (2012) demonstrated that calcium added to soil as CaCl_2_ helped to raise the total protein content by 1.7% in chickpea seeds (*C. arietinum* L.) compared to seeds obtained from plants growing in unlimed soil. The results reported by Datta et al. (2012) from an experiment on the same plant demonstrated that the total protein content was 92% lower in treatments polluted with the dose of 4 mM of fluorine compared to the treatment unpolluted with this element.

## Conclusions

The effect of increasing soil contamination with fluorine on the yields and content of selected nitrogen forms in the test plants depended on a plant species and organ, dose of fluorine added to soil, and the applied substance inactivating fluorine. In general, the soil pollution with fluorine stimulated the biomass of the test plants. However, there were exceptions, for example, the amount of biomass of spring triticale grain and straw, maize roots, and lucerne aerial parts decreased in response to the soil pollution with fluorine. The plant species were characterized by different degrees of tolerance to soil contamination with fluorine. Among the plants tested in our experiment, lucerne was most sensitive to fluorine in soil, even though smaller doses of this xenobiotic were used in this series. The other plant species were quite tolerant to an elevated content of fluorine in soil. Out of the three examined neutralizing substances, lime was most effective in alleviating the negative effect of soil contamination with fluorine on the harvested biomass of the test plants; charcoal was slightly less effective while loam was least effective in this regard. Under the influence of the growing soil pollution with fluorine, the analyzed organs of most of the test plants contained more total nitrogen, with the highest accumulation was found in both organs of black radish, grain, and roots of spring triticale and aerial biomass of yellow lupine. A decrease in the N-total content in response to the soil pollution with fluorine was noticed only in the aerial biomass of yellow lupine. With respect to protein nitrogen, an increase in its content occurred in grain of spring triticale and in roots of black radish, while the aerial biomass of winter oilseed rape contained less of this nitrogen form. In most of the test plants, the soil contamination with fluorine caused an increase in the content of N-NO_3_^−^ in commercially usable plant organs. Out of the three tested neutralizing substances, loam had a distinctly negative effect on this form of nitrogen.

## References

[CR1] Asthir B, Basra AS, Batta SK (1999). Differential response of carbon and nitrogen metabolism to fluoride application in fruiting structures of chickpea (*Cicer arietinum*). Acta Physiol Plant.

[CR2] Bhargava D, Bhardwaj N (2010). Effect of sodium fluoride on seed germination and seedling growth of *Triticum aestivum VAR. RAJ. 4083*. J Phytol.

[CR3] Chakrabarti S, Patra PK, Mandal B, Mahato D (2012). Effect of sodium fluoride on germination, seedling growth and biochemistry of bengal gram *(Cicer arietinum)*. Fluoride.

[CR4] Cui X, Wang XD, Fan WH, Wang JM, Cui KY (2011). Effects of fluoride on soil properties and yield and quality of maize. Chin J Eco-Agric.

[CR5] Datta JK, Maitra A, Mondal NK, Banerjee A (2012). Studies on the impact of fluoride toxicity on germination and seedling growth of gram seed *(Cicer arietinum L. cv. Anuradha)*. J Stress Physiol Biochem.

[CR6] Dey U, Mondal NK, Das K, Datta JK (2012). Dual effects of fluoride and calcium on the uptake of fluoride, growth physiology, pigmentation and biochemistry of bengal gram seedlings (*Cicer arietinum* L.). Fluoride.

[CR7] Elloumi N, Abdallah BF, Mezghani I, Rhouma A, Boukhris M (2005). Effect of fluoride on almond seedlings in culture solution. Fluoride.

[CR8] Elrashidi MA, Persaud N, Baliger VC (1998). Effect of fluoride and phosphate on yield and mineral composition of barley grown on three soils. Commun Soil Sci Plan.

[CR9] Eyini M, Sujanandini K, Pothiraj C, Jayakumar M, Kil B-S (1999). Differental response of *Azolla microphylla* Kaulf. and *Azolla filiculoides* Lam. to sodium fluoride. J Plant Biol.

[CR10] Gadi BR, Verma P, Amra R (2012). Influence of NaF on seed germination, membrane stability and some biochemicals content in Vigna seedlings. J Chem Biol Phys Sci.

[CR11] Gupta S, Banerjee S, Mondal S (2009). Phytotoxity of fluoride in the germination of paddy *(Oryza sativa)* and its effect on the physiology and biochemistry of germinated seedlings. Fluoride.

[CR12] Holopainen JK, Kainulainen E, Oksanen J, Wulff A, Kärenlampi L (1991). Effect of exposure to fluoride, nitrogen compounds and SO_2_ on the numbers of spruce shoot aphids on Norway spruce seedlings. Oecologia.

[CR13] Jha SK, Nayak AK, Sharma YK, Mishra VK, Sharma DK (2008). Fluoride accumulation in soil and vegetation in the vicinity of brick fields. Bull Environ Contam Toxicol.

[CR14] Joshi M, Bhardwaj N (2012). Effect of fluoride on growth parameters and its accumulation in *Triticum aestivum var. Raj. 3675*. Fluoride.

[CR15] Kabata-Pendias A, Pendias H (1999) Biogeochemistry of trace elements. PWN-Warsaw, pp 398 (in Polish)

[CR16] Kumar S, Singh M (2015). Effect of fluoride contaminated irrigation water on eco-physiology, biomass and yield in *Gossypium hirsutum* L. Trop Plant Res.

[CR17] Nowak J, Kuran B, Smolik B (2000) Dynamics of fluorine conversion from soluble forms into insoluble water compounds in various soil. Folia Univ Agric Stetin 209 Agricultura 83:125–130

[CR18] Ochoa-Herrera V, Banihani Q, Leon G, Khatri C, Fidel AJ, Sierra-Alvarez R (2009). Toxicity of fluoride to microorganisms in biological wastewater treatment systems. Water Res.

[CR19] Ostrowska A, Gawliński S, Szczubiałka Z (1991). Methods for analyses and assessment of soil and plant properties.

[CR20] Pal CK, Mondal KN, Bhaumik R, Banerjee A, Datta KJ (2012). Incorporation of fluoride in vegetation and associated biochemical changes due to fluoride contamination in water and soil: a comparative field study. Ann Environ Sci.

[CR21] Pant S, Pant P, Bhiravamurthy PV (2008). Effects of fluoride on early root and shoot growth of typical crop plants of India. Fluoride.

[CR22] Rao BVA, Kumar AK, Nagalaksmamma K, Vidyunmala S (2013). Effect of fluoride on protein profiles in two cultivars of mulberry leaves. AE J Agri Envir Sci.

[CR23] Rey-Asensio A, Carballeia A (2007). *Lolium perenne* as a biomonitor of atmospheric levels of fluoride. Environ Inter.

[CR24] Saini P, Khan S, Baunthiyal M, Sharma V (2012). Organ-wise accumulation of fluoride in *Prosopis juliflora* and its potential for phytoremediation of fluoride contaminated soil. Chemosphere.

[CR25] Saleem M, Ahmad NM, Khan AB, Zia A, Ahmad SS, Shah UH, Khan AN, Qazi MI (2015). Effects of soil fluoride on the growth and quality of two tomato varieties grown in Peshawar, Pakistan. Fluoride.

[CR26] Smolik B, Telesiński A, Szymczak J, Zakrzewska H (2011). Assessing of humus usefulness in limiting of soluble fluoride content in soil. Ochr Środ Zas Nat.

[CR27] StatSoft Inc. (2010) STATISTICA data analysis software system, version 10.0.www.statsoft.com

[CR28] Stevens DP, McLaughlin MJ, Alston AM (1998). Phytotoxicity of hydrogen fluoride and fluoroborate and their uptake from solution culture by *Lycopersicon esculentum* and *Avena sativa*. Plant Soil.

[CR29] Stevens DP, McLaughlin MJ, Alston AM (1998). Phytotoxicity of the fluoride ion and its uptake from solution culture by *Avena sativa* and *Lycopersicon esculentum*. Plant Soil.

[CR30] Telesiński A, Śnioszek M (2009). Bioindicators of environmental pollution with fluorine. Brom Chem Toksykol.

[CR31] Telesiński A, Siwczyk F, Zakrzewska H (2012). An attempt to determination of the 50% phytotoxicity threshold for different fluoride concentrations affecting the spring wheat (*Triticum aestivum* L.) and white mustard (*Sinapis alba* L.) seedlings. Fluoride.

[CR32] Weinstein HL, Davison AW (2003). Native plant species suitable as bioindicators and biomonitors for airborne fluoride. Environ Pollut.

[CR33] Wulff A, Kärenlampi L (1996). Effects of long-term open-air exposure to fluoride, nitrogen compounds and SO_2_ on visible symptoms, pollutant accumulation and ultrastructure of Scots pine and Norway spruce seedlings. Trees.

[CR34] Yamauchi M, Yoshida H, Taniyama T, Umezaki T, Nagaya Y (2000). Remove from marked records: effects of fluorine added to irrigation water at various concentrations under soil culture on the growth of rice plants. Japan J Crop Sci.

[CR35] Yu MH (1996). Effects of fluoride on growth and soluble sugars in germinating mung bean *(Vigna radiate)* seeds. Fluoride.

[CR36] Zhang L, Li Q, Ma L, Ruan J (2013). Characterization of fluoride uptake by roots of tea plants (*Camellia sinensis* L. O. Kuntze). Plant Soil.

